# Left ventricular myocardial deformation assessment in asymptomatic patients with recently diagnosed sarcoidosis of the respiratory tract and/or extrapulmonary sarcoidosis

**DOI:** 10.1186/s13023-021-02038-2

**Published:** 2021-09-29

**Authors:** Roman Panovský, Martina Doubková, Mary Luz Mojica-Pisciotti, Tomáš Holeček, Jan Máchal, Věra Feitová, Lucia Masárová, Lukáš Opatřil, Vladimír Kincl, Jana Víšková

**Affiliations:** 1 International Clinical Research Center, St. Anne’s Faculty Hospital, Faculty of Medicine, Masaryk University, Brno, Czech Republic; 2 1St Department of Internal Medicine and Cardioangiology, St. Anne’s Faculty Hospital, Faculty of Medicine, Masaryk University, Pekarska 53, 656 91 Brno, Czech Republic; 3 Department of Pulmonary Diseases and Tuberculosis, Faculty of Medicine and University Hospital, Masaryk University, Brno, Czech Republic; 4 Department of Medical Imaging, St. Anne’s Faculty Hospital, Brno, Czech Republic; 5 Department of Pathophysiology, Faculty of Medicine, Masaryk University, Brno, Czech Republic

**Keywords:** Cardiac magnetic resonance, Sarcoidosis, Feature tracking, Strain analysis

## Abstract

**Background:**

Sarcoidosis is a systemic granulomatous disease affecting different organs including the heart. Myocardial strain analysis could potentially detect the early stages of cardiac dysfunction in sarcoidosis patients. The present study aims to assess the use of cardiac magnetic resonance (CMR) strain analysis using feature tracking (FT) in the detection of early cardiac involvement in asymptomatic patients with sarcoidosis.

**Methods:**

One hundred and thirteen CMR studies of patients with sarcoidosis of the respiratory tract and/or extrapulmonary sarcoidosis without pre-existing known cardiovascular disea*se* were included in the study and analysed using FT and compared to 22 age and gender-matched controls. Global longitudinal strain (GLS), global circumferential strain (GCS) and global radial strain (GRS) of the left ventricle (LV) were measured.

**Results:**

The sarcoidosis patients did not significantly differ from the controls in basic demographic data and had normal global and regional systolic LV function—LV ejection fraction (EF) 66 ± 7% vs 65 ± 5% in the controls (*p* = NS). No statistically significant differences were found in all strain parameters between patients and controls: GLS (− 13.9 ± 3.1 vs. − 14.2 ± 2.5), GCS (− 23.4 ± 4.0 vs. − 22.2 ± 2.9) and GRS (53.4 ± 13.5 vs. 51.2 ± 13.6%) (*p* = NS).

**Conclusion:**

Patients with sarcoidosis of the respiratory tract and/or extrapulmonary sarcoidosis had normal myocardial deformation measured by CMR-FT derived global strain

## Background

Sarcoidosis is a rare systemic granulomatous disease of unknown cause. Most frequently, the lungs and lymph nodes are involved, but also other organs, including the heart, can be affected. Clinically evident sarcoidosis involves the heart in 2–7% of patients, but occult involvement is much higher—even 30–40% according to autopsy and/or imaging studies [1–4]. Granulomatous inflammation may involve any part of the heart. The most typical clinical symptoms of cardiac sarcoidosis are arrhythmias (20–30%), especially atrioventricular block and ventricular arrhythmias, but the clinical status can vary from asymptomatic to syncope or low left ventricular (LV) ejection fraction (EF) with congestive heart failure or even to sudden cardiac death [5–7]. Consequently, especially with regard to the risk of sudden death and worse prognosis of patients with cardiac sarcoidosis and reduced LV EF, the early diagnosis of cardiac involvement could be beneficial for patients.

The diagnosis of cardiac sarcoidosis is very challenging. Cardiac magnetic resonance imaging (CMR), together with Holter monitoring, echocardiography, and nuclear techniques, has become a major pillar in the diagnostic process [8–10]. Late gadolinium enhancement (LGE) and LV systolic function are the most significant decision-making parts of the CMR examination. Nevertheless, global LV EF and visible regional wall motion abnormalities may not be sensitive enough to detect incipient subtle changes in LV function. Besides exact global and regional functional assessment, and imaging of structural changes in the myocardium (granulomas and inflammation), CMR could offer a relatively new technique of myocardial strain evaluation. Myocardial deformation could be affected earlier by slight, incipient pathological changes in the myocardium [11–16].

Myocardial strains can be measured by various echocardiographic and CMR techniques. Echocardiography has an advantage over CMR because of its higher frame rate but is often limited by the image quality. On the other hand, CMR is restricted by lower phases, although, it profits from better imaging of the epicardial-myocardial and endocardial-myocardial tissue boundaries. CMR feature tracking (FT) is one of the myocardial deformation imaging techniques that has been tested for several clinical conditions, even in patients with preserved LV EF [11, 17–19]. Nevertheless, there is very little evidence for CMR-FT in patients with recent sarcoidosis [20].

## Methods

This study aims to assess the use of CMR-FT derived myocardial strain in the detection of early cardiac involvement in asymptomatic patients with sarcoidosis.

### Patient population and CMR data acquisition

Patients with sarcoidosis of the respiratory tract and/or extrapulmonary sarcoidosis and available CMR examination were enrolled into this retrospective study. Patients with Löfgren syndrome were excluded. Patients with any pre-existing known cardiovascular disease or cardiac symptoms or any signs of cardiac involvement of sarcoidosis prior to screening were also excluded. A control group was introduced as a group of people with unknown or by routine examination including CMR undetectable heart disease, typically examined for any suspicion that has not been confirmed.

CMR examinations were performed in a single-center using a 3.0 T MR Discovery 750 scanner (GE Healthcare, Chicago, United States). The detailed scanning protocol was published previously [21] and included a balanced steady-state gradient echo sequence (Fast Imaging Employing Steady-state Acquisition—FIESTA) in a short axis stack and three long-axis; a T2-weighted sequence (Triple Inversion Recovery Fast Spin Echo); native and postcontrast MOLLI (Modified Look-Locker inversion recovery); and SMART_1_Map (Saturation method using adaptive recovery times for cardiac T1 mapping) sequences, and LGE images.

The study was performed in accordance with the Declaration of Helsinki (2000) of the World Medical Association. The presented analysis is the retrospective one of a previously published study that was approved by the institutional ethics committee (Ethics Committee of St Anne’s University Hospital Brno, reference number 65V/2015) [21]. Written informed consent was obtained from the subjects and/or their legally authorized representative.

### CMR data analysis

Myocardial strain analysis was performed using the CMR-FT method by the commercially available software Image Arena (2D CPA MR, version 4.6.4.40, TomTec Imaging Systems GmbH, Unterschleissheim, Germany). A detailed description of the methodology has been described our previous studies [22, 23]*.* Analyses were done in a random and blinded order regarding the patient clinical characteristics. Both the endocardial and epicardial contours were manually traced in long-axis (2, 3 and 4-chamber views) and short-axis images (basal, midventricular and apical levels) during end-diastole and end-systole. The manually traced contours were propagated throughout the images for the complete cardiac cycle. The global LV strains were automatically measured and calculated as the mean of the segmental strain values. Short-axis images were used to determine the global circumferential strain (GCS) and global radial strain (GRS), while long-axis ones (2, 3 and 4-chamber views) were used for the global longitudinal strain (GLS).

To assess the interobserver and intraobserver agreement, as well as precision, 10 random patients were blindly evaluated by two experienced observers (M.M. and T.H.), one of them performed the analysis twice.

### Statistical analysis

The Student t-test was used to compare continuous variables in patients and controls, which generally followed the Gaussian distribution as assessed by the Kolmogorov–Smirnov test and visual inspection of the histograms. The same method was also used for subgroup analysis (comparing patients with and without LGE, or those with and without corticosteroid treatment); one-way ANOVA with Tukey post-hoc test was employed for more than two groups. The Fisher exact test was used for the comparison of binary variables. For the eventual association of the pulmonary sarcoidosis stage with the strain values, the Spearman correlation coefficients with respective *p* values were determined, while the association with ejection fraction was expressed using the Pearson correlation coefficient.

Intraobserver and interobserver agreement was assessed using the intraclass correlation coefficient (ICC). The precision of the method was evaluated as the mean standard deviation per case (three measurements, 10 cases). The α = 0.05 cut-off was used throughout the analysis. All analyses were performed using Statistica (version 13.5. TIBCO Software Inc., 2018) software.

## Results

One hundred and thirteen CMR studies of patients with known sarcoidosis of the respiratory tract and/or extrapulmonary sarcoidosis and without pre-existing known cardiovascular disea*se* were included in the study. Because of significant imaging artefacts in cine images, three patients were not suitable for detailed strain analysis. One hundred and ten studies of sarcoidosis patients and 22 healthy controls were analysed using CMR-FT. The characteristics of both groups are shown in Table 1. Not surprisingly, sarcoidosis patients used corticosteroids more often (57% vs. 5%; *p* < 10–5); they also had a higher body mass index (BMI; 29.1 ± 4.4 vs. 26.4 ± 4.7; *p* = 9.10–3), which was likely due to corticosteroid treatment, and suffered more from dyspnoea (60% vs. 5%; *p* < 10–5), most likely due to the main pulmonary diagnosis. The other baseline characteristics, including other medication and LVEF, did not differ between the groups. No patient had angina pectoris.

**Table 1:** Basic characteristics of the study groups

	Sarcoidosis n = 113	Control group n = 22	*p* value
Age [years]	52.0 ± 10.8	52.7 ± 11.1	0.78
Female [n (%)]	51 (45.1%)	8 (36.4%)	0.49
**BMI [kg/m**^**2**^**]**	**29.1 ± 4.4**	**26.4 ± 4.7**	**9.10**^**–3**^
**Dyspnoea [n (%)]**	**58 (60.2%)**	**1 (4.6%)**	** < 10**^**–5**^
Hypertension [n (%)]	35 (31.0%)	7 (31.8%)	1.00
Diabetes [n (%)]	6 (5.3%)	0 (0.0%)	0.59
**Corticosteroids [n (%)]**	**64 (56.6%)**	**1 (4.6%)**	** < 10**^**–5**^
ACE-inhibitors [n (%)]	14 (12.4%)	4 (18.2%)	0.49
ARBs [n (%)]	13 (11.5%)	0 (0.0%)	0.13
β-blockers [n (%)]	16 (14.2%)	1 (4.6%)	0.31
Diuretics [n (%)]	5 (4.4%)	1 (4.6%)	1.00
Statins [n (%)]	11 (9.7%)	1 (4.6%)	0.69
LV EF [%]	66 ± 7	65 ± 5	0.29

Variables marked in bold have *p*-value < 0.05

Continuous variables are expressed as the mean ± standard deviation, binary variables as count (percentage). The *p* values refer to the *t* test in the case of continuous variables or to the Fisher exact test in binary variables

*BMI* body mass index, *ACE* angiotensin converting enzyme, *ARBs* angiotensin receptor blockers, *LV* left ventricle, *EF* ejection fraction

At the time of CMR examination, the mean time from the diagnosis of sarcoidosis was 0.8 (0.2–3.3) years. Most of the patients were in stage 2 according to their X-ray findings (52%) and 30% of the patients also had extrapulmonary sarcoidosis (in the nodes, spleen, eyes, skin, liver, bones, epididymis or neuro-sarcoidosis). All sarcoidosis patients had preserved global systolic LV function (LV EF 66 ± 7% vs 65 ± 5% in controls, *p* = NS) without any regional hypokinesia or akinesia. No patient had any signs of myocardial edema according to T2-weighted images. Questionable small LGE was found in 8 sarcoidosis patients (7%), however the LGE patterns were more or less atypical from the viewpoint of expected LGE in cardiac sarcoidosis. As published previously, the comparison of global native T1 values, post-contrast T1 values and ECV values did not show any difference between the patients and controls using either SMART1Map or MOLLI sequence.

**Fig. 1:**
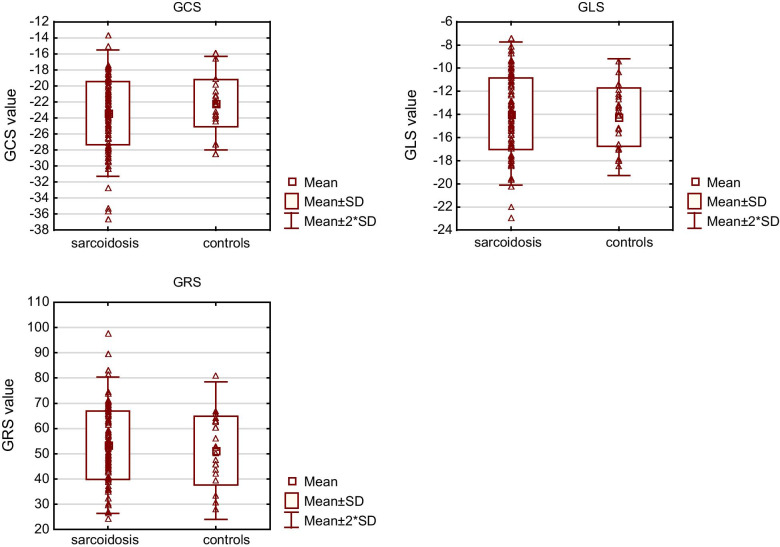
Global strain values in sarcoidosis patients and controls

The results of global strain measurement are shown in Table 2 and Fig. 1. There were no significant differences between the groups. In a detailed analysis, there was no correlation of global strains with the pulmonary sarcoidosis stage (all *p* > 0.10, all r between − 0.15 and 0.15). All global strains correlated with the ejection fraction in the sarcoidosis patients (GLS: r = − 0.42, *p* < 0.001; GCS: r = − 0.65, *p* < 0.001; GRS: 0.31, *p* = 0.001). In all cases, the absolute value of the strains were positively correlated with the EF (GLS and GCS were expressed as negative numbers). However, correlations were similar in the control subjects (GLS: r = − 0.18, *p* < 0.42; GCS: r = − 0.70, *p* < 0.001; GRS: 0.38; *p* = 0.08) and there was no significant difference between correlations in the sarcoidosis patients and controls (GLS: *p* = 0.29; GCS: *p* = 0.71; GRS: *p* = 0.75). The data for strain values in the sarcoidosis patients divided into quartiles according to EF are shown in Table 3.

**Table 2:** Comparison of global left ventricular strain

	Sarcoidosis n = 110	Control group n = 22	*p* value
GLS (%)	− 13.9 ± 3.1	− 14.2 ± 2.5	0.68
GCS (%)	− 23.4 ± 4.0	− 22.2 ± 2.9	0.16
GRS (%)	53.4 ± 13.5	51.2 ± 13.6	0.49

Variables with the Gaussian distribution are expressed as the mean ± standard deviation

*GLS* global longitudinal strain, *GCS* global circumferential strain, *GRS* global radial strain

**Table 3:** Comparison of global left ventricular strain in sarcoidosis patients by quartiles of ejection fraction

	Q1	Q2	Q3	Q4	*p* value
GLS (%)	− 12.1 ± 3.9	− 13.6 ± 2.4	− 14.2 ± 2.8	− 15.6 ± 2.9	1.10^–5^
GCS (%)	− 19.7 ± 3.1	− 22.3 ± 2.1	− 23.7 ± 2.5	− 26.2 ± 4.3	2.10^–9^
GRS (%)	− 43.6 ± 9.7	54.1 ± 11.2	55.4 ± 14.4	56.6 ± 14.3	0.081

Variables with the Gaussian distribution are expressed as the mean ± standard deviation

*GLS* global longitudinal strain, *GCS* global circumferential strain, *GRS* global radial strain

Subgroup analyses further revealed no association of global strains with LGE positivity (GLS: − 13.9 ± 3.1 in LGE negative vs. − 14.0 ± 3.2 in LGE positive, *p* = 0.96; GCS: − 23.3 ± 3.9 in LGE negative vs. − 24.6 ± 4.8 in LGE positive, *p* = 0.36; GRS: 53.7 ± 13.7 in LGE negative vs. 49.5 ± 11.0 in LGE positive, *p* = 0.57), corticosteroid treatment (GLS: − 14.1 ± 3.0 in patients without corticoids vs. − 13.8 ± 3.2 in patients on corticoids, *p* = 0.71; GCS: − 24.2 ± 4.0 in patients without corticoids vs. − 22.8 ± 3.9 in patients on corticoids, *p* = 0.07; GRS: 54.7 ± 14.5 in patients without corticoids vs. 52.3 ± 12.7 in patients on corticoids, *p* = 0.38), or the presence or localization of extrapulmonary sarcoidosis (all *p* > 0.10, data not shown).

The global strain measurement showed a very good intraobserver agreement (ICC 0.859 (GLS), 0.966 (GRS), 0.953 (GCS)) and interobserver agreement (ICC 0.848 (GLS), 0.919 (GRS), 0.904 (GCS)). At the same time, precision was as follows: GLS: 0.78%; GRS: 4.93%; and GCS: 0.90%.

## Discussion

To the best of our knowledge, this is so far the largest study using CMR-FT derived myocardial strain in patients with sarcoidosis. The main finding is the fact that asymptomatic patients with recent extracardiac sarcoidosis had normal myocardial deformation measured by CMR-FT derived global LV strain.

In contrast with the relatively extensive literature using echo-derived strain analysis, there are only two published articles using CMR-derived strain assessment in sarcoidosis patients. Dabir et al. [20] investigated 61 patients with biopsy-proven sarcoidosis using CMR-FT. GLS was significantly reduced in patients with sarcoidosis, even with an otherwise inconspicuous CMR, such that GLS may serve as a marker for early cardiac involvement. On the other hand, circumferential strain parameters were reduced only in patients with other CMR signs of cardiac sarcoidosis. In comparison to our study, there was a longer time since the diagnosis (≥ 10 years in 18% of patients) as well as a significantly higher percentage of LGE positive patients (70%) compared to our cohort (7%).

In the second manuscript, a different CMR technique, tagging imaging, was used for only circumferential strain analysis in 13 cardiac sarcoidosis patients [24]. Circumferential strain and strain rate were better in LGE negative segments than in LGE positive ones. No comparison with a control group was done, and neither any global strain analysis nor longitudinal data were described.

Several other groups have studied echo-derived strain using 2D or 3D speckle tracking echocardiography (STE) [25–30]. From the results, GLS has seemed to be the best marker. Almost all papers described reduced LV GLS, even in patients with a normal LV EF and no wall motion abnormalities [25, 27, 28]. On the contrary, the usefulness of radial and circumferential strain is questionable as the results are inconsistent. Echo-derived GLS could also serve as a prognostic marker, as sarcoidosis patients with reduced GLS have been associated with future adverse events including cardiovascular death, cardiac dysfunction, high-grade atrioventricular block, or malignant ventricular arrhythmia [31–36]. STE has been considered an exact and suitable technique to assess myocardial strain. It provides non-Doppler, angle-independent, and objective quantification of myocardial deformation. However, its widespread applicability may be complicated by poor acoustic windows. It is the main reason for the development of several CMR techniques for assessing strain. Generally, GLS seemed to be more exact with STE, while GCS showed better reproducibility if calculated with CMR-FT. Among strain values assessed with CMR-FT, GRS has large ranges between studies [18, 37, 38]. All tracking techniques have proved to be more robust and reproducible for global strain values when compared to regional ones [38, 39].

There is no easy explanation of the finding of normal global LV strain of sarcoidosis patients in our study in the light of other trials. Presumably, the main reason could be the fact that the population was examined in a very recent stage of the disease. There are several indicators for this postulate. First, the time since diagnosis was shorter (0.8; 0.2–3.3 years) in comparison to other studies. Second, LGE was observed in 7% of patients, while others found between 25 and 70% of patients with LGE [20, 40, 41]. Likewise, no patient in our study had any signs of myocardial edema on T2-weighted images, which was also unexpected. Furthermore, as previously published [21], myocardial native T1 relaxation times were not prolonged, and the extracellular volume (ECV) was not increased in the cohort. It seems that at the very beginning of the disease, the myocardium could remain unaffected.

Despite the study results, CMR-FT presents a very appropriate method, that could be a part of a multiparametric examination of patients at risk of cardiac sarcoidosis. Along with heart function assessment, signs of edema, imaging of granulomas by LGE, T1 relaxation time and ECV, myocardial strain analysis could bring detailed information about potential myocardial involvement. In contrast to some published papers that combined echo-derived strain with LGE [26], CMR offers a “one-stop” approach obtaining an extensive spectrum of data. There is also a potential for monitoring of the therapy using CMR-derived strain [42]. However, based on the results of this study, CMR should not become the first-line examination in patients with diagnosed sarcoidosis who have no clinical suspicion of cardiac involvement.

The study has several limitations. No endomyocardial biopsy (EMB) as a standard for cardiac involvement was performed. Nevertheless, EMB is an invasive technique that is not currently clinically performed in such patients and only very rarely used for research purposes, as its accuracy is not optimal. The results may be potentially affected by local process settings and center-specific bias, as this is a single-center study. Also, segmental strain data were not used for the analysis as CMR-FT does not provide sufficient reproducibility for using them now. Potential regional abnormalities could be missed due to this fact.

Also important are the differences of previously reported normal ranges of CMR-FT based strain analysis. For instance, Taylor et al. [12] reported substantially different normal ranges of CMR-FT in comparison to our controls. Nevertheless, they described normal values for myocardial strain in healthy subjects (GLS mean value was − 19.1 ± 4.1%) from the analyses of images acquired in a 1.5 T scanner (Magnetom Avanto, Siemens, Erlangen, Germany), while, in our study (GLS mean value was − 14.2 ± 2.5%), subjects were examined using a 3.0 T MR Discovery 750 scanner (GE Healthcare, Chicago, USA). Similarly, Wang et al. [43] used the same scanner as us and reported a GLS of − 13.55 ± 3.28% for healthy volunteers. So, normal values for GLS probably depend on many parameters, including the vendor and the software. To explain the discrepancy between the GLS values, several technical points should be considered. The difference in the magnetic field affects the SSFP images, making them more sensitive to susceptibility and banding artefacts [44, 45], therefore, impacting the image quality. Likewise, no patient-specific frequency adjustments to mitigate off-resonance effects were performed. The endocardial layer positioning also has a quantifiable influence on strain measurements [46]. Using the Image Arena software, the contours were located at the inside frontier of the blood pool, which is the most typical positioning, and after contouring the epicardial wall, the software applied the tracking algorithm. The contrast to noise ratio of the images during this process was not changed to avoid adding variability to the analysis because it cannot be modified equally in all the images. In any case, our work does not aim to suggest reference values for this sarcoidosis population or healthy controls.

## Conclusion

Asymptomatic patients with a recent diagnosis of sarcoidosis of the respiratory tract and/or extrapulmonary sarcoidosis had normal myocardial deformation measured by CMR-FT derived global strain.

## Data Availability

The datasets analyzed during the current study are available from the corresponding author upon reasonable request.

## Abbreviations

ACE: Angiotensin converting enzyme
ARBs: Angiotensin receptor blockers
BMI: Body mass index
CMR: Cardiac magnetic resonance
ECV: Extracellular volume
EF: Ejection fraction
EMB: Endomyocardial biopsy
FIESTA: Fast Imaging Employing Steady-state Acquisition
FT: Feature tracking
GCS: Global circumferential strain
GLS: Global longitudinal strain
GRS: Global radial strain
ICC: Intraclass correlation coefficient
LGE: Late gadolinium enhancement
LV: Left ventricle
MOLLI: Modified Look-Locker inversion recovery
SMARTMap_1_: Saturation method using adaptive recovery times for cardiac T1 mapping
STE: Speckle tracking echocardiography
T: Tesla
